# Global Mental Health Research: Time to Integrate Basic Science

**DOI:** 10.1007/s11469-021-00625-9

**Published:** 2021-09-13

**Authors:** Susan M. Meffert

**Affiliations:** Department of Psychiatry, San Francisco (UCSF), University of California, San Francisco, CA USA

**Keywords:** Global mental health, Research, Translational research, Neuroscience, Implementation science, Neurobiology, Funding priorities, NIMH, Basic science, COVID, Lancet Commission

## Abstract

The Lancet Commission on Global Mental Health recommends the inclusion of research on the biological underpinnings of mental disorders as part of efforts to reduce the global burden of mental disorders. The search for defining features of mental disorders in non-Euro American settings is historically charged for the field. Yet, as illustrated by analysis of the NIMH objectives, the biological study of mental disorders cannot be scientifically sound without better inclusion of under-represented, globally diverse populations. It is time for global mental health researchers to take up the challenge and advance impactful research across the full translational spectrum.

With an escalating global health burden of mental disorders (James et al., 2018),scientific communities today are facing urgent needs to (1) identify the biological underpinnings of mental disorders, biomarkers, and new treatment targets, and (2) identify strategies to scale up existing evidence-based treatments to address the massive dearth of mental health care, particularly in resource-poor settings. These research areas are traditionally construed as existing at different ends of a translational research “pipeline,” which can lead to competing claims for priority in research funding and departmental, institutional, and academic attention. In this commentary, conventional “silos” of what is often termed “global mental health (GMH)” research and neuroscientific investigations are examined and the value of new approaches is considered, using NIMH research priorities for illustration.

Beginning in 2007, the Lancet series on Global Mental Health initially focused on downstream translational research—particularly treatment implementation needs—and served as a formative call to action for mental health professionals, including clinicians, public health programs, and clinical researchers (Lancet Global Mental Health Group, 2007). One of the key drivers of the first publication was recognition of the massive worldwide burden of mental disorders, highlighted by the Global Burden of Disease Studies. A 75% “treatment gap”—the proportion of individuals who have a serious mental disorder (such as major depression) and do not receive treatment—was underscored in the 2007 publication. The second Lancet series (2011) (Patel et al., 2011) highlighted vulnerable populations in need of mental health research to inform services (e.g., children and adolescents, people in humanitarian emergencies), described the global human resource gaps, and explored the intersections of human rights, poverty, and mental disorders.

In a largely separate process, ambitions to understand the human brain prompted global brain initiatives to harmonize and integrate interdisciplinary work across the research spectrum. For example, in 2013, President Obama launched the U.S. Brain Research through Advancing Innovative Neurotechnologies (BRAIN) Initiative. Also in 2013, the European Union launched the Human Brain Initiative to integrate brain science, medicine, and computing. The Japanese government-initiated Brain/MINDS (Brain Mapping by Integrated Neurotechnologies for Disease Studies) in 2014. In Australia, the Brain Initiative was launched and in Korea a 10-year effort to map the brain is underway. These efforts, many of which emphasize basic neuroscientific research, are crucial to advancing our understanding of mental disorders.

The 2018 Lancet Commission on Global Mental Health and Sustainable Development moved beyond service provision (Patel et al., 2018). While persisting with efforts to address the treatment gap, the Commissioners recommended that the field of GMH also focus on the convergence of biological and social research to identify the determinants of mental health for disease prevention and reduction of population burden. Biological and genetic risk factors for mental disorders were specifically cited by the commission as important topics of investigation. As one of the rallying points for the burgeoning GMH research community, Lancet’s recommendation signals an important opportunity for mental health researchers across the translational spectrum.

In 2020, the need for collaboration between neuroscientific and GMH implementation research priorities gained vivid illustration with the Coronavirus Disease 2019 (COVID-19) pandemic. COVID not only challenges mental health research communities to quickly grasp the pathophysiology of neuropsychiatric sequelae for prevention and treatment, but also to rapidly develop strategies for expanded, remote care access to address the colossal COVID-related increase of depression and anxiety disorders across the globe.

## Mental Disorder and Etiology: Neuroscience and Global Mental Health

Tension between universal and local sources of mental disorders may have discouraged neuroscientific investigations in GMH research. The history of GMH research includes long debates to accurately and ethically negotiate between universal and biological understandings of mental disorders versus those that are indexed to local culture (Ommeren et al., 2005; Summerfield, 1982). The field of medical anthropology was an important contributor to these debates. Dr. Arthur Kleinman, a psychiatrist and medical anthropologist, used the concept of the “category fallacy” and argued that the cultural context of emotional experience is central to the definition of mental conditions as normal or pathological (Kleinman, 1977). The World Health Organization (WHO) led a number of early studies exploring mental disorders and outcomes across multiple countries. One of these was the International Pilot Study of Schizophrenia, which followed the course of 1202 individuals diagnosed with schizophrenia in nine countries and reported heterogeneous outcomes between countries of low and high economic development (Sartorius et al., 1972). The WHO was also an early investigator of strategies to diagnose mental disorders in different cultural settings (Sartorius et al., 1980).

Today, the effort to optimally define mental pathology continues, now with a focus on creating integrated understandings that embrace both biological and environmental variables. Research organizations, including the NIMH and the European Roadmap for Mental Health Research, created initiatives to explore pathways that cut across current diagnostic categories, such as the Research Domain Criteria (RDoC) (Marquand et al., 2016). Deploying a powerful set of new technologies, researchers strive to disaggregate and reorganize biological and environmental factors into categories that more consistently define mental health and dysfunction. However, most of these studies focus on populations in high-income countries and have not included the global diversity of mental disorder manifestations (Hyman, 2019). Endeavors to define the underpinnings of mental disorders are unlikely to succeed unless we can find a way to ethically investigate them across a broad range of human genetic and environmental influences. Traditional divisions between GMH and neuroscientific research obstruct our advancement toward better understanding and treatment of mental disorders. Below, the NIMH strategic priorities are explored as an example of how cooperation between GMH and neuroscience research could accelerate science toward its goals.

## Objectives in Mental Health Research

NIMH Strategic Objective 1, “Define the Mechanisms of Complex Behaviors,” includes studies on genetics and gene-environment interplay in the mechanism of mental disorders. Without a global diversity of genetic and environmental factors in our study populations, we risk selection bias and compromised external validity. Scientific recognition of the need for this genetic diversity is exemplified by the Broad Institute’s Neuropsychiatric Genetics in African Populations (NeuroGAP) study (Hyman, 2019).

Epidemiologist Sandro Galea describes the importance of robust investigations of gene-environment interplay. Galea asserts that epidemiology should expand its focus on causal architecture to include the “exposure-based epidemiologies”—the macrosocial, economic, and political determinants of health and disease (Galea, 2019; Galea & Keyes, 2019). Galea argues that the mechanisms of mental health and dysfunction will never be coherent or effective for the improvement of population health unless we grapple concurrently with both the biological and the macrosocial determinants of disease. Because we cannot study the effect of macrosocial determinants of disease without a diversity of those exposures, it follows that we cannot effectively study gene-environment interplay without inclusion of populations living with a global diversity of social, economic, and political conditions.

NIMH Strategic Objective 2, “Chart Mental Illness Trajectories To Determine When, Where, and How to Intervene,” includes observational research to identify developmental trajectories of the brain and behavior across the lifespan, as well as clinically useful biomarkers to predict illness change. Observational research is sensitive to misclassification and selection bias. Misclassification refers to an information bias (such as culturally irrelevant mental disorder definitions) resulting in misdiagnosis—studying mental disorders across a wide range of cultures improves the chances of identifying those universal, biological factors that contribute to mental illness across all populations. Selection bias refers to failure to select a representative sample (e.g., failing to include a global diversity of study populations), limiting the identification of biological drivers of mental disorders and the generalizability of study results. Without a wide diversity of biological, social, and other environmental factors, as well as diverse life courses in the study population, we risk selection bias and compromised external validity, restricting the advance of mental health research and related population health gains in the USA.

GMH treatment research continues to be pertinent to NIMH research Objectives 3 (Prevention and Cure) and 4 (Strengthen Public Health Impact). Expanded focus on prevention in GMH research would bolster NIMH Objective 3 by including a full array of biological, social, and other environmental determinants to promote efficient identification of modifiable risk (prevention) factors for mental disease. Finally, NIMH Objective 4 is one of the fortes of GMH research. Researchers in diverse settings find it essential to maximize their public health impact and define it in terms that are clear and persuasive for policy makers to implement the research findings and achieve public health impact.

## Conclusions

GMH research appears not only compatible with neuroscientific research, but necessary to advance biologic understandings of mental disorders by including the vast diversity of populations with whom the field engages. Certainly, basic science mental health studies with vulnerable populations must be thoughtfully and collaboratively integrated with implementation research and have clear, short-term benefit for the community. However, with its long history of ethical and anthropological debate, the GMH community is well-equipped to successfully meet these challenges. The Lancet commission offers an exciting vision of how we might more intentionally bring basic and implementation science researchers together under the umbrella GMH. Our current COVID pandemic provides a pressing forum to do so.
